# Prediction model of in-hospital mortality in intensive care unit patients with cardiac arrest: a retrospective analysis of MIMIC -IV database based on machine learning

**DOI:** 10.1186/s12871-023-02138-5

**Published:** 2023-05-25

**Authors:** Yiwu Sun, Zhaoyi He, Jie Ren, Yifan Wu

**Affiliations:** 1 Department of Anesthesiology, Dazhou Central Hospital, No.56 Nanyuemiao Street, Tongchuan District, Dazhou, Sichuan 635000 China; 2 Department of Anesthesiology, The Third Affiliated Hospital of Harbin Medical University, No.150 Haping Road, Nangang District, Harbin, Heilongjiang 150000 China; 3 Department of Anesthesiology, Guizhou Provincial People’s Hospital, No.83 Zhongshan East Road, Nanming District, Guiyang, Guizhou 550002 China; 4 Department of Anesthesiology, Shanghai Sixth People’s Hospital, No.600 Yishan Road, Xuhui District, Shanghai, 200030 China

**Keywords:** Prediction model, Machine learning, Cardiac arrest, Intensive care unit, In-hospital mortality, MIMIC-IV database

## Abstract

**Background:**

Both in-hospital cardiac arrest (IHCA) and out-of-hospital cardiac arrest (OHCA) have higher incidence and lower survival rates. Predictors of in-hospital mortality for intensive care unit (ICU) admitted cardiac arrest (CA) patients remain unclear.

**Methods:**

The Medical Information Mart for Intensive Care IV (MIMIC-IV) database was used to perform a retrospective study. Patients meeting the inclusion criteria were identified from the MIMIC-IV database and randomly divided into training set (*n* = 1206, 70%) and validation set (*n* = 516, 30%). Candidate predictors consisted of the demographics, comorbidity, vital signs, laboratory test results, scoring systems, and treatment information on the first day of ICU admission. Independent risk factors for in-hospital mortality were screened using the least absolute shrinkage and selection operator (LASSO) regression model and the extreme gradient boosting (XGBoost) in the training set. Multivariate logistic regression analysis was used to build prediction models in training set, and then validated in validation set. Discrimination, calibration and clinical utility of these models were compared using the area under the curve (AUC) of the receiver operating characteristic (ROC) curves, calibration curves and decision curve analysis (DCA). After pairwise comparison, the best performing model was chosen to build a nomogram.

**Results:**

Among the 1722 patients, in-hospital mortality was 53.95%. In both sets, the LASSO, XGBoost,the logistic regression(LR) model and the National Early Warning Score 2 (NEWS 2) models showed acceptable discrimination. In pairwise comparison, the prediction effectiveness was higher with the LASSO,XGBoost and LR models than the NEWS 2 model (*p* < 0.001). The LASSO,XGBoost and LR models also showed good calibration. The LASSO model was chosen as our final model for its higher net benefit and wider threshold range. And the LASSO model was presented as the nomogram.

**Conclusions:**

The LASSO model enabled good prediction of in-hospital mortality in ICU admission CA patients, which may be widely used in clinical decision-making.

**Supplementary Information:**

The online version contains supplementary material available at 10.1186/s12871-023-02138-5.

## Background

The morbidity and mortality surrounding CA remained a global challenge. In the United States, more than 350,000 people experience an OHCA every year, and about 10.8% of them survive to hospital discharge [1]. The in-hospital survival rate of OHCA patients was 26.4% [2]. IHCA occurs in over 290,000 adults each year in the United States [3]. The 30 day survival rate of patients with IHCA was 27.8%, and the one-year survival rate was 20% [4]. Despite increasing attention to CA, the prognosis of patients with CA was still unsatisfactory.

Most patients with CA died in acute events. There were also some deaths in patients with CA that occur after successful resuscitation, which can be attributed to the development of post CA syndrome, including neurological dysfunction and other types of organ dysfunction. For patients with successful resuscitation, they will generally be sent to the intensive care unit (ICU) for advanced life support treatment. About one third of patients admitted to the ICU survive to discharge, but there are considerable differences in the treatment and prognosis of patients after CA between different institutions [5–7]. Given the high hospital mortality rate, identifying high-risk factors and accurately predicting prognosis in the early stages of hospitalization may have greater benefits for patients with cardiac arrest admitted to the ICU. Although there were several models for predicting mortality in hospitalized patients with CA available, the accuracy of these methods was not satisfactory (the sample size was less than 1000 or the C-statistic was not calculated), so they had not been widely used [8–10].

Machine learning (ML) belongs to the category of artificial intelligence [11]. Different from the traditional prediction model that uses selected variables for calculation, ML can not only easily combine a large number of variables with computers to improve the accuracy of prediction, but also screen variables by a variety of methods when selecting variables to improve the accuracy and efficiency of the model [12].

By using ML to screen variables and establish prediction models, adverse factors for patients with CA can be identified at the early stage of admission to the ICU, and corrected as soon as possible to improve the prognosis of patients. The purpose of this study was to develop and validate a predictive model for in-hospital mortality of patients with CA admitted to ICU using data from MIMIC-IV database.

## Materials and methods

### Study design and data source

We conducted a retrospective analysis using all the relevant data extracted from the MIMIC-IV database.

The MIMIC-IV database is an open and publicly available database that contains high-quality data between 2008 and 2019 constructed by Institutional Review Boards of the Massachusetts Institute of Technology (MIT, Cambridge, MA, America) and Beth Israel Deaconess Medical Center. To access the database, we passed the National Institutes of Health Protecting Human Research Participants web-based training course and we obtained approval to extract data from the MIMIC-IV for research purposes (Certification Number: 50778029).

### Study patients

**Fig. 1:**
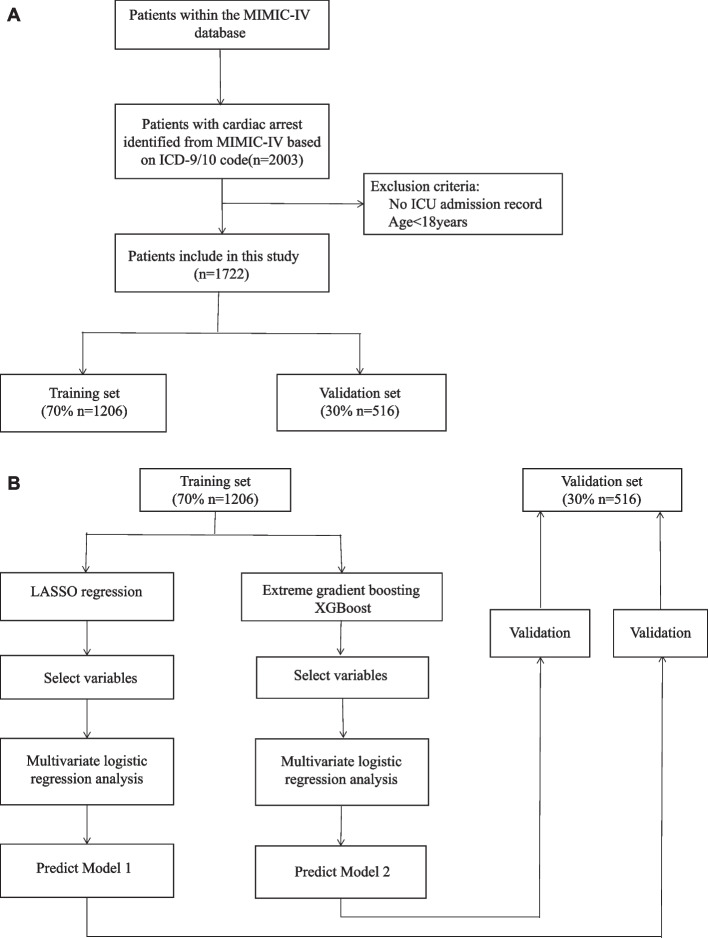
**A** Flowchart of patient selection (*n* = 1722). ICD-9/10, 9/10th revision of the International Classification of Diseases; ICU, intensive care unit; MIMIC-IV, Medical Information Mart for Intensive Care IV. **B** Model development flowchart. LASSO, least absolute shrinkage and selection operator; XGBoost, extreme gradient boosting

Patients with a diagnosis of CA, defined as ICD-9 codes of 4275 or ICD-10 codes of I46, I462, I468 and I469. Patients were ≥ 18 years old at the time of ICU admission were included in the study; Patients without an ICU record were excluded from the study. The flow chart showed the selection of patients into the study (Fig. 1A). Patients with a diagnosis of CA were screened and 1722 adult patients were included in this study.

### Data extraction and processing

Demographics, vital signs, laboratory tests, scoring systems, relevant treatment information, and others were extracted from the MIMIC-IV database using structured query language with PostgreSQL (version 14, www.postgresql.org). The prediction model only included the clinical and laboratory variables on the first day of ICU admission. If the patient received more than one vital sign measurement or laboratory tests on the first day of admission, the average values were used for subsequent analysis. Comorbidities were identified using ICD-9/10 code. Based on previous research [8, 9, 13, 14], clinical relevance, and general availability, the following data were extracted: demographic characteristics (age at the time of hospital admission, sex); vital signs (heart rate (HR), systolic blood pressure (SBP), diastolic blood pressure (DBP), mean blood pressure(MBP), respiratory rate(RR), body temperature, saturation pulse oxygen (SPO_2_); comorbidities (hypertension, congestive heart failure(CHF), myocardial infarction, diabetes mellitus(DM), and chronic obstructive pulmonary disease (COPD)) and laboratory variables (hematocrit(HCT), hemoglobin(HB), platelet count, white blood cells(WBC), prothrombin time (PT), international normalized ratio (INR), creatinine, blood urea nitrogen (BUN), glucose, potassium, sodium, calcium, chloride, the anion gap, bicarbonate, lactate, hydrogen ion concentration (pH); treatment information(ventilation, epinephrine, dopamine); marking system: sequential organ failure assessment(SOFA), simplified acute physiology score III (SAPS III), Glasgow coma scale (GCS). The primary outcome of the study was in-hospital mortality, defined as the vital status of the patient at discharge.

### Missing data handling

Variables with missing data are common in the MIMIC-IV, and directly eliminating patients with missing values or analyzing variables with missing values will cause bias. we excluded variables with more than 25% of missing values.

For variables with missing proportion < 5%, the continuous variables with normal distribution, the missing values were replaced with the mean for the patient group; The skewed distributions with continuous variables, missing values were replaced with their median [15]. Multiple imputation can impute each missing value with multiple plausible possible values. This method takes into account uncertainty behind the missing value and can produce several datasets from which parameters can be estimated, and these coefficients are combined to give an effective estimate of the coefficients [16–19]. For variables with missing proportion > 5%, we used multiple imputation to handle the data by R software ('mice' package). (Missing value details in Supplementary Table 1).

### Statistical analysis

Values were presented as total numbers with percentages for categorical variables and the means with standard deviations (SD) (if normal) or medians with inter quartile ranges (IQR) (if non normal) for continuous variables. Proportions were compared using χ^2^ test or Fisher exact tests. For all continuous variables, we used a two-sided one-way analysis of variance or Wilcoxon rank-sum tests when comparing the two groups. *P* < 0.05 was statistically significant.

The flow chart demonstrates the methodology for developing the predictive model (Fig. 1B). According to previous research [20], the data were divided at random, with 70% utilized for training and 30% for validating. Table 1 summarized the predictor variables and statistics.

**Table 1:** Characteristics of participants

	**Total (*n* = 1722)**	**Training set (*n* = 1206)**	**Validation set (*n* = 516)**	***P* value for Training vs validation**	**Alive (*n* = 793)**	**Died (n = 929)**	***P* value for Alive vs died**
Age (years old) (mean (SD))	66.76 (16.61)	66.92 (16.61)	66.37 (16.62)	0.527	65.14 (16.35)	68.14 (16.71)	< 0.001
Man (n%)	1033 (59.99)	705 (58.46)	328 (63.57)	0.054	504 (63.56)	529 (56.94)	0.006
SBP (mmHg) (mean (SD))	113.43 (17.87)	112.62 (17.53)	115.31 (18.51)	0.004	116.93 (15.52)	110.44 (19.17)	< 0.001
DBP (mmHg) (mean (SD))	62.20 (12.16)	61.75 (11.90)	63.24 (12.71)	0.019	63.48 (10.79)	61.10 (13.12)	< 0.001
MBP (mmHg) (mean (SD))	76.69 (12.26)	76.21 (12.14)	77.82 (12.47)	0.012	78.77 (10.47)	74.92 (13.36)	< 0.001
Heart rate (beats/minute) (mean (SD))	85.00 (18.43)	85.20 (18.45)	84.53 (18.37)	0.488	80.87 (16.36)	88.52 (19.35)	< 0.001
Respiratory rate(beats/minute) (mean (SD))	20.92 (4.43)	20.93 (4.42)	20.90 (4.46)	0.885	19.80 (4.04)	21.87 (4.53)	< 0.001
Temperature (°C) (mean (SD))	36.44 (1.16)	36.46 (1.15)	36.41 (1.19)	0.407	36.68 (0.90)	36.24 (1.31)	< 0.001
^SPO^2 ^(%) (mean (SD))^	96.18 (5.20)	96.05 (5.60)	96.50 (4.10)	0.099	97.39 (2.19)	95.15 (6.61)	< 0.001
**Comorbidities, n (%)**							
Hypertension (n, %)	661 (38.39)	458 (37.98)	203 (39.34)	0.632	316 (39.85)	345 (37.14)	0.270
Myocardial infarction (n, %)	416 (24.16)	297 (24.63)	119 (23.06)	0.526	211 (26.61)	205 (22.07)	0.033
Congestive heart failure (n, %)	396 (23.00)	280 (23.22)	116 (22.48)	0.787	204 (25.73)	192 (20.67)	0.015
Diabetes mellitus (n, %)	263 (15.27)	186 (15.42)	77 (14.92)	0.848	109 (13.75)	154 (16.58)	0.119
Chronic obstructive pulmonary disease (n, %)	129 (7.49)	92 (7.63)	37 (7.17)	0.817	70 (8.83)	59 (6.35)	0.064
**Laboratory parameters**							
Anion gap (mmol/L) (median (IQR))	16.50 (14.00–20.00)	16.50 (14.00, 20.00)	16.50 (13.88, 19.50)	0.541	15.00 (13.50, 18.00)	17.50 (15.00, 22.00)	< 0.001
BUN (mg/dL) (median (IQR))	25.00 (17.00–41.00)	25.00 (17.00, 40.50)	25.00 (17.00, 42.62)	0.695	21.50 (15.00, 34.50)	29.00 (19.00, 46.00)	< 0.001
Bicarbonate (mmol/L) (median (IQR))	21.50 (18.00–24.50)	21.00 (18.00, 24.50)	21.50 (18.00, 25.00)	0.099	23.00 (20.00, 25.00)	19.50 (16.50, 23.00)	< 0.001
Creatinine (mg/dL) (median (IQR))	1.35 (0.90–2.20)	1.35 (0.90, 2.19)	1.35 (0.90, 2.16)	0.966	1.15 (0.85, 1.80)	1.50 (1.00, 2.45)	< 0.001
Chloride(mmol/L) (median (IQR))	104.0 (99.50–107.50)	104.00 (100.00, 107.50)	103.59 (99.50, 107.50)	0.388	104.00 (100.00, 107.00)	104.00 (99.50, 108.00)	0.158
Glucose (mg/dL) (median (IQR))	161.00 (125.50–214.00)	161.00 (126.00, 215.50)	161.00 (124.50, 211.12)	0.655	148.00 (120.50, 191.50)	170.50 (133.50, 234.00)	< 0.001
Hematocrit (%)	33.49 (6.98)	33.49 (7.10)	33.51 (6.71)	0.950	34.00 (6.57)	33.06 (7.29)	0.006
Hemoglobin (g/dL) (mean (SD))	10.89 (2.38)	.90 (2.40)	10.87 (2.33)	0.817	11.18 (2.28)	10.64 (2.44)	< 0.001
INR (median (IQR))	1.33 (1.15–1.75)	1.33 (1.15, 1.75)	1.33 (1.15, 1.80)	0.814	1.25 (1.10, 1.45)	1.45 (1.20, 2.10)	< 0.001
PT(s) (median (IQR))	14.70 (12.80–19.10)	14.70. (12.80, 18.95)	14.70 (12.75, 19.23)	0.868	13.70 (12.25, 16.20)	15.70 (13.55, 22.50)	< 0.001
Lactate (mmol/L) (median (IQR))	3.30 (1.80–5.60)	3.26 (1.80, 5.60)	3.30 (1.80, 5.56)	0.999	2.46 (1.60, 4.30)	4.18 (2.20, 6.70)	< 0.001
Platelet (109 /L) (median (IQR))	193 (142.00–257.50)	189.75 (140.00, 255.12)	199.00 (146.38, 268.50)	0.104	196.50 (151.50, 257.00)	189.00 (129.00, 258.50)	0.007
pH (median (IQR))	7.32 (7.24–7.39)	7.32 (7.24, 7.39)	7.34 (7.24, 7.39)	0.210	7.35 (7.28, 7.40)	7.30 (7.21, 7.38)	< 0.001
Potassium (mmol/L) (mean (SD))	4.39 (0.73)	4.39 (0.73)	4.39 (0.74)	0.930	4.33 (0.64)	4.44 (0.80)	0.003
Sodium (mmol/L) (mean (SD))	138.68 (5.29)	138.67 (5.24)	138.70 (5.39)	0.915	138.36 (4.47)	138.95 (5.88)	0.020
Calcium (mmol/L) (mean (SD))	8.38 (1.13)	8.39 (1.17)	8.37 (1.02)	0.718	8.47 (0.85)	8.31 (1.32)	0.003
WBC (109 /L) (median (IQR))	12.55 (9.05–17.05)	12.55 (9.15, 17.20)	12.53 (8.84, 16.70)	0.483	11.75 (8.75, 15.60)	13.20 (9.50, 18.20)	0.003
Dopamine (n, %)	283 (15.97)	208 (17.25)	68 (13.18)	0.042	94 (11.85)	182 (19.59)	< 0.001
Epinephrine (n, %)	359 (20.26)	242 (20.07)	100 (19.38)	0.794	103 (12.99)	239 (25.73)	< 0.001
Ventilation (n, %)	1526 (88.61)	1068 (88.56)	458 (88.76)	0.969	710 (89.53)	816 (87.84)	0.303
**Scoring systems**							
SOFA (mean (SD))	8.65 (4.53)	8.85 (4.52)	8.40 (4.58)	0.056	7.34 (4.47)	9.89 (4.26)	< 0.001
SAPS III (mean (SD))	70.57 (30.63)	70.94 (30.74)	70.82 (30.85)	0.937	59.06 (28.58)	81.02 (28.91)	< 0.001
GCS (mean (SD))	14.00 (2.78)	14.04 (2.72)	13.92 (2.91)	0.429	14.56 (1.31)	13.53 (3.51)	< 0.001
In-hospital death (n, %)	929 (53.95)	673 (55.80)	256 (49.61)	0.021			

Data are presented as %, mean ± SD or median (IQR)

*BUN* Blood urea nitrogen, *DBP* Diastolic blood pressure, *GCS* Glasgow coma scale, *HB* Hemoglobin, *INR* International normalized ratio, *MBP* Mean blood pressure, *PH* Hydrogen ion concentration, *PT* Prothrombin time, *SAPS III* Simplified Acute Physiology Score III, *SBP* Systolic blood pressure, *SOFA* Sequential organ failure assessment, *SPO2* Saturation pulse oxygen, *WBC* White blood cell. *P* < 0.05 was statistically significant

Two ML methods were used to select the most important predictors of the in-hospital mortality prediction model from the training set. First, we used the LASSO method, which was conducted via a continuous shrinking operation and minimizing regression coefficients, in order to reduce the likelihood of overfitting. And, LASSO can shrink the sum of the absolute value of regression coefficients, forcing and producing coefficients that are exactly 0 [21, 22]. Variables with non-zero coefficients were selected for the next logical regression analysis. This enhanced the prediction accuracy and interpretation ability of the prediction model, and which was suitable for high-dimensional data processing.

Second, we used XGBoost, which was an efficient and scalable ML classifier. XGBoost can achieve high prediction accuracy and low computational costs in various practical applications [23]. Gradient boosting decision tree is the original model of XGBoost, which combines multiple decision trees in boosting way. XGBoost used the number of boosts, learning rate, subsampling ratio, and maximum tree depth to control overfitting and enhance the better performance. Moreover, XGBoost optimized the target of the function, the size of the tree, and the size of the weight through regularization [24].

To investigate the independent risk factors of in-hospital mortality, the variables screened by two methods were used in the training set of univariate logistic regression analysis to evaluate the significance of variables. In the univariate logistic regression analysis, the variables significantly related to the in-hospital mortality will be further analyzed by multivariate logistic regression analysis. Prediction models were evaluated in terms of discrimination and calibration. Discrimination was assessed by calculating the area under the curve (AUC) of the receiver operating characteristic (ROC) curve and C-statistic testing. Decision curve analysis (DCA) was used to compare the clinical net benefit associated with the use of these models [25].

The model with the highest AUC and the highest clinical net benefit was identified as the final model, and a nomogram was drawn to predict in-hospital mortality. The nomogram was a visualization of the results of the regression equation by calculating the probability of occurrence by using some disjoint segments.

The National Early Warning Score 2(NEWS2) was a modification of NEWS and a simple aggregate scoring system. In the early stage of disease deterioration, the NEWS2 can detect the potential disease changes in patients early and provide preventive measures for disease deterioration, which has important clinical implications to improve the survival rate of patients with CA in ICU [26–30]. Because the final calculation was a score, we performed subsequent analysis after translating it into a univariate predict model, to analyze C-index and estimate differences in the discrimination between the models.

Meanwhile, we used a stepwise method to construct a prediction model in logical regression(LR model).

All analyses were performed by the statistical software packages R version 4.2.2 (http://www.R-project.org, The R Foundation). *P* < 0.05 (two-sided test) were considered statistically significant.

## Result

### Baseline characteristics

As shown in Fig. 1A, a total of 1722 patients diagnosed as CA were included in our study. According to the research method of the previous study, we randomly divided all patients into training set (1206 people, 70%) and validation group (516 people, 30%). In the whole study population, the in-hospital mortality rate of CA patients was 53.95% (793 survivors and 929 non-survivors). Table 1 showed the comparison of demographics and variables between the training set and the validation set, as well as the comparison of dead patients and survivors during hospitalization. SBP, DBP, MBP values were lower in the training set. The proportion of dopamine use and in-hospital mortality were lower in the validation set. There were no significant differences in other selected variables between the training set and validation set. Patients who died also had lower SBP, DBP, MBP, temperature, SPO_2_, HCT, HB, platelet, bicarbonate, calcium, pH, GCS score, the proportion of man, CHF and myocardial infarction. However, age, SOFA score and SAPS III score, epinephrine use, dopamine use, HR, RR, WBC, anion gap, BUN, creatinine, glucose, sodium, potassium, INR, PT and lactate levels in patients who died during their hospital stay were significantly increased. There was no significant difference in terms of whether they had DM, hypertension, ventilation and chloride levels, between the surviving and non-surviving patients.

### Selected variables

**Fig. 2:**
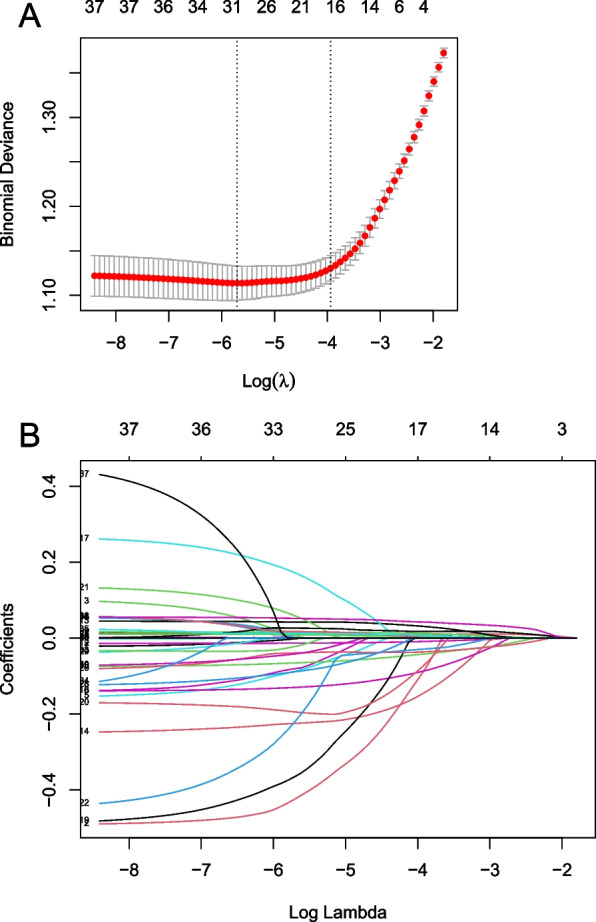
**A** Cross validation plot for the penalty term. 17 potential prediction variables were selected. **B** A coefficient profile plot was produced against the Log Lambda sequence

**Fig. 3:**
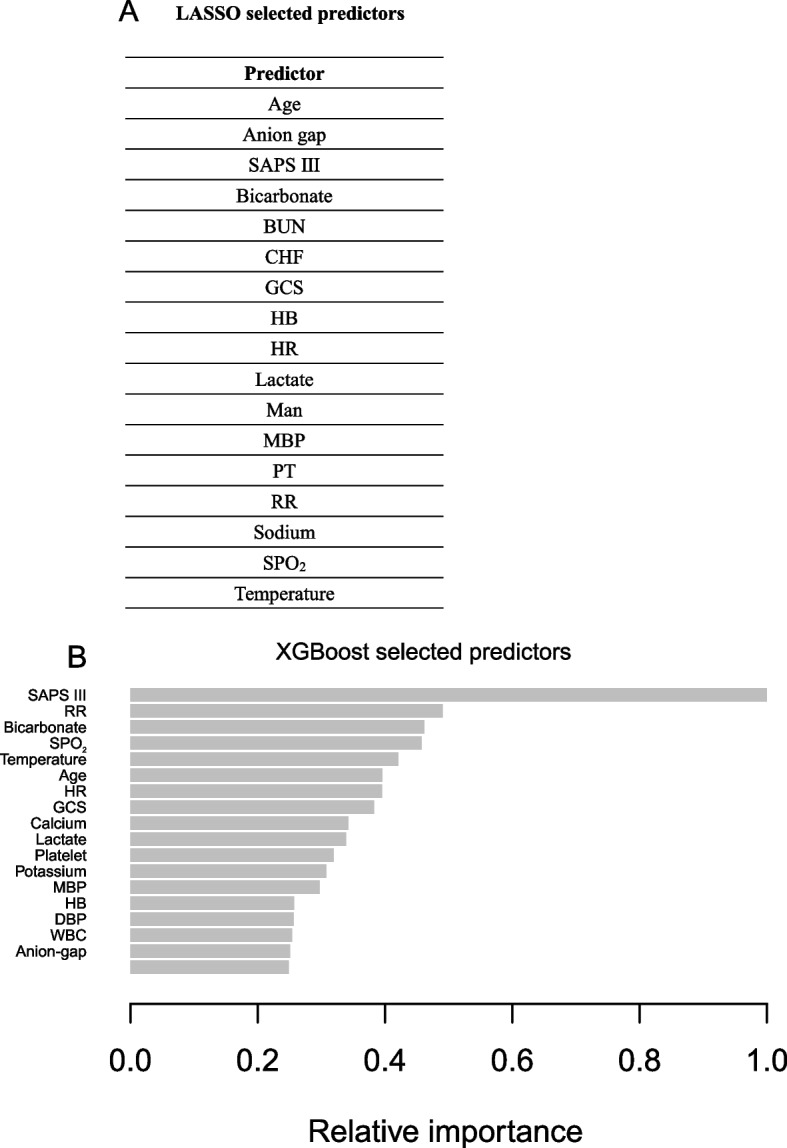
Predictor variables selection. **A** Predictor variables selected by LASSO. **B** Importance ranking of the predictor variables selected by XGBoost algorithm. BUN, blood urea nitrogen; CHF, congestive heart failure; DBP, diastolic blood pressure; GCS, Glasgow coma scale; HB, hemoglobin; HR, heart rate; LASSO, least absolute shrinkage and selection operator; MBP, mean blood pressure; PT, prothrombin time; RR, respiratory rate; SAPS III, simplified acute physiology score; SPO2, saturation pulse oxygen; WBC, white blood cells; XGBoost, extreme gradient boosting

In the training set, we conducted the regularization process of LASSO. The binomial deviance was computed for the test data as measures of the predictive performance of the fitted models. The binomial deviance curve was plotted versus log (λ) using tenfold cross-validation via minimum criteria, where λ was a tuning hyperparameter. The dotted vertical lines were drawn at optimal values by using the minimum criteria and within one standard error range of the minimum criteria. We chose the latter criteria (λ = 0.01944) as it results in stricter penalty allowing us to reduce the number of covariates even further than the minimum criteria (λ = 0.00332) (Fig. 2A, B). Finally, 17 nonzero coefficients were resulted in LASSO regression (Fig. 3A). Meanwhile, XGboost was also used to analyze the patients who died in the training set, ranked the predictive importance of all included variables, and selected the top 17 variables (Fig. 3B).

### Model development

In the training set, 17 variables respectively screened by LASSO and XGboost were used to conduct univariate logistic regression with the in-hospital mortality, and variables with statistically significance in univariate logistic regression were used to conduct multivariate logistic regression. Tables 2 and 3 showed the variables selected in the univariate and multivariate analysis by LASSO and XGBoost. Among the variables screened using LASSO, multivariate logistic regression identified age, SAPS III, HR, MBP, RR, temperature, SPO2, GCS, man, bicarbonate, PT as the most significant mortality risk predictors; Among the XGBoost selected variables, SAPS III, RR, bicarbonate, SPO_2_, temperature, age, HR, GCS, HB as the most significant mortality risk predictors.

**Table 2:** Univariate and multivariate logistic regression analyze variables screened by least absolute shrinkage and selection operator regression in the training set

	Univariate analysis			Multivariate analysis		
	OR	95%CI	P	OR	95%CI	
Age (years old)	1.01	1.01 to 1.02	< 0.001	1.01	1.00 to 1.02	0.013
Congestive heart failure	0.79	0.61 to 1.04	0.0928	NA		
SAPS III	1.02	1.02 to 1.03	< 0.0001	1.01	1.00 to 1.01	0.002
Heart rate (beats/minute)	1.03	1.02 to 1.03	< 0.001	1.02	1.01 to 1.03	< 0.001
MBP (mmHg)	0.97	0.96 to 0.98	< 0.001	0.99	0.98 to 1.00	0.049
Respiratory rate (beats/minute)	1.13	1.09 to 1.16	< 0.001	1.05	1.01 to 1.09	0.007
Temperature (°C)	0.72	0.64 to 0.80	< 0.001	0.76	0.65 to 0.87	< 0.001
SPO_2_ (%)	0.86	0.82 to 0.90	< 0.001	0.93	0.88 to 0.97	0.002
GCS	0.85	0.80 to 0.90	< 0.001	0.86	0.80 to 0.91	< 0.001
Man (n, %)	0.73	0.58 to 0.92	0.0084	0.76	0.57 to 1.00	0.048
Hemoglobin (g/dL)	0.89	0.85 to 0.94	< 0.001	0.95	0.89 to 1.01	0.100
Anion gap (mmol/L)	1.14	1.11 to 1.18	< 0.001	1.02	0.97 to 1.06	0.466
Bicarbonate (mmol/L)	0.89	0.86 to 0.91	< 0.001	0.95	0.92 to 0.98	0.003
BUN (mg/dL)	1.02	1.01 to 1.02	< 0.001	1.01	1.00 to 1.01	0.087
Sodium (mmol/L)	1.02	1.00 to 1.04	0.0702	NA		
PT (s)	1.05	1.03 to 1.07	< 0.001	1.01	1.00 to 1.03	0.049
Lactate (mmol/L)	1.25	1.19 to 1.31	< 0.001	1.06	0.99 to 1.13	0.103

*BUN* Blood urea nitrogen, *GCS* Glasgow coma scale, *MBP* Mean blood pressure, *PT* Prothrombin time, *SAPS III* Simplified acute physiology score, *SPO*_*2*_ Saturation pulse oxygen. *P* < 0.05 was statistically significant

**Table 3:** Univariate and multivariate logistic regression analyze variables screened by extreme gradient boosting in the training set

	Univariate analysis			Multivariate analysis		
	OR	95%CI	P	OR	95%CI	P
SAPS III	1.02	1.02 to 1.03	< 0.001	1.01	1.00 to 1.02	0
SPO_2_ (%)	0.86	0.82 to 0.90	< 0.001	0.92	0.88 to 0.96	0.001
Age (years old)	1.01	1.01 to 1.02	< 0.001	1.01	1.00 to 1.02	0.005
Respiratory rate (beats/minute)	1.13	1.09 to 1.16	< 0.001	1.05	1.01 to 1.09	0.009
Lactate (mmol/L)	1.25	1.19 to1.31	< 0.001	1.05	0.99 to 1.12	0.096
Bicarbonate (mmol/L)	0.89	0.86 to 0.91	< 0.001	0.96	0.93 to 0.99	0.023
Heart rate (beats/minute)	1.03	1.02 to 1.03	< 0.001	1.02	1.01 to 1.03	< 0.001
Platelet (10^9^ /L)	1.00	1.00 to 1.00	0.1729	NA		
Temperature (°C)	0.72	0.64 to 0.80	< 0.001	0.77	0.66 to 0.88	< 0.001
Calcium (mmol/L)	0.90	0.80 to 0.99	0.0347	0.93	0.82 to 1.07	0.308
GCS (mmol/L)	0.85	0.80 to 0.90	< 0.001	0.86	0.81 to 0.92	< 0.001
Potassium (mmol/L)	1.22	1.04 to 1.43	0.0157	0.93	0.76 to 1.13	0.441
Anion gap (mmol/L)	1.14	1.11 to 1.18	< 0.001	1.04	1.00 to 1.08	0.051
MBP (mmHg)	0.97	0.96 to 0.98	< 0.001	0.98	0.96 to 1.01	0.217
Hemoglobin (g/dL)	0.89	0.85 to 0.94	< 0.001	0.92	0.86 to 0.98	0.008
WBC (10^9^ /L)	1.02	1.01 to 1.04	0.0022	1.00	0.99 to 1.02	0.665
DBP (mmHg)	0.98	0.97 to 0.99	< 0.001	1.00	0.98 to 1.03	0.903

*DBP* Diastolic blood pressure, *GCS* Glasgow coma scale, *MBP* Mean blood pressure, *SAPS III* Simplified acute physiology score, *SPO*_*2*_ Saturation pulse oxygen, *WBC* White blood cells. *P* < 0.05 was statistically significant

We established an in-hospital mortality prediction algorithm using LASSO selected variables as follows: log odds of mortality = 18.746877 + 0.013344 × age + 0.010997 × SAPS III + 0.019006 × HR- 0.017839 × MBP + 0.048912 × RR- 0.286264 × temperature- 0.080727 × SPO_2_- 0.142085 × GCS- 0.258837 × man- 0.064604 × bicarbonate + 0.021723 × PT.

The variance inflation factors for these variables were 1.1, 1.2, 1.3, 1.1, 1.3, 1.2, 1.1, 1.0, 1.0, 1.1 and 1.0, respectively.

Based on XGBoost, the selected variables for the in-hospital mortality prediction algorithm were as follows: log odds of mortality = 20.258476 + 0.011845 × SAPS III + 0.052959 × RR- 0.071588 × bicarbonate- 0.099384 × SPO_2_- 0.279351 × temperature + 0.013794 × age + 0.018419 × HR- 0.138026 × GCS- 0.102837 × HB.

The variance inflation factors for these variables were 1.2, 1.3, 1.1, 1.1, 1.2, 1.1, 1.3, 1.0 and 1.1, respectively.

Based on stepwise logistic regression, the selected variables for the in-hospital mortality prediction algorithm were as follows: log odds of mortality = 16.287329 + 0.013572 × age- 0.447131 × CHF + 0.010371 × SAPSIII + 0.018552 × HR- 0.016852 × MBP + 0.049526 × RR- 0.286100 × temperature- 0.086035 × SPO_2_- 0.135393 × GCS- 0.530986 × COPD + 0.137646 × HCT- 0.461777 × HB- 0.067713 × bicarbonate + 0.007738 × BUN + 0.025168 × sodium.

The variance inflation factors for these variables were 1.2, 1.2, 1.3, 1.3, 1.2, 1.3, 1.2, 1.1, 1.0, 1.1, 13.4, 13.8, 1.2, 1.2 and 1.1, respectively.

### Model validation

**Fig. 4:**
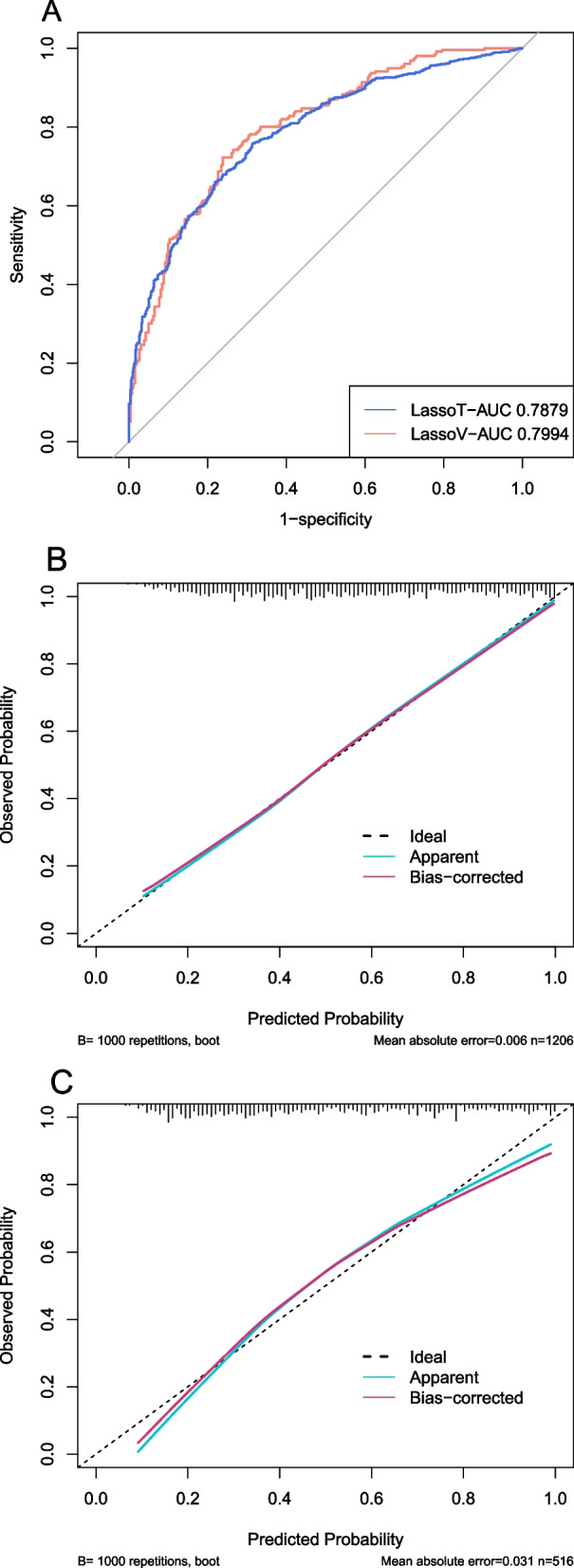
The discrimination and calibration performance of LASSO model. Plot (**A**) showed the ROC curves of the LASSO model in the training set and validation set, respectively (AUC = 0.7879 versus 0.7994). Calibration curves of the LASSO model in the training set (**B**) and validation set (**C**). Calibration curves depicted the calibration of the LASSO model in terms of the agreement between the predicted risk of in-hospital mortality and observed in-hospital mortality. The 45° dotted line represents a perfect prediction, and the blue lines represent the predictive performance of the LASSO model. The closer the violet line fit is to the ideal line, the better the predictive accuracy of the LASSO model is. AUC, area under the curve; LASSO, least absolute shrinkage and selection operator; ROC, receiver operating characteristic

**Fig. 5:**
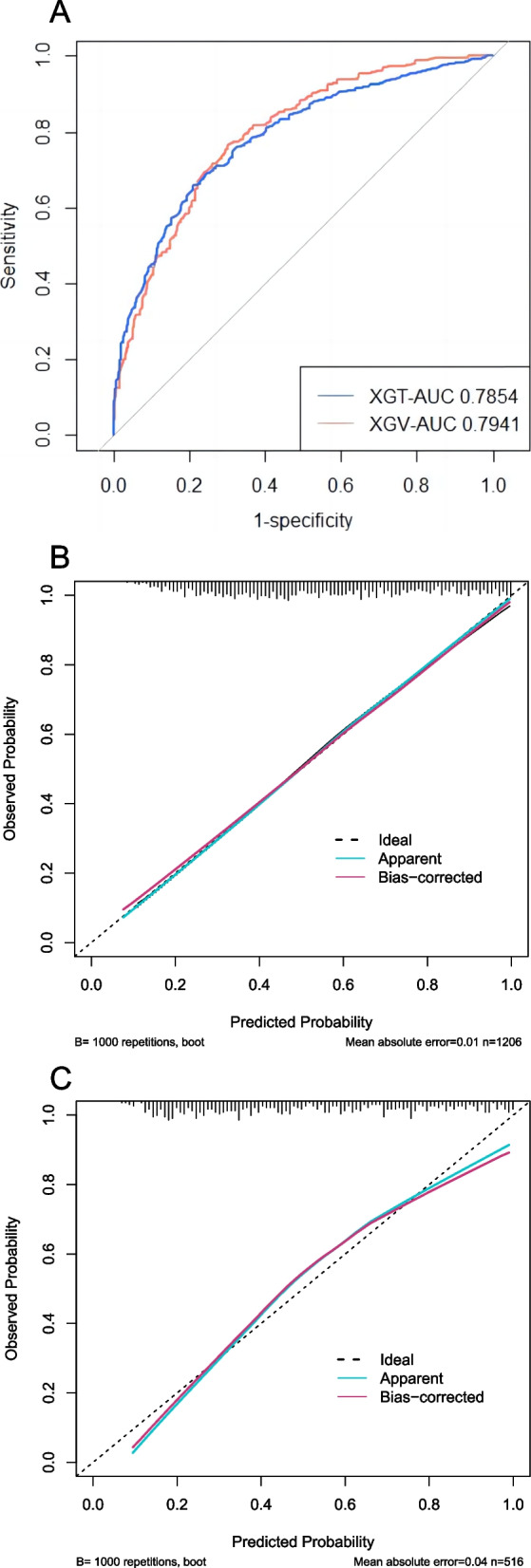
The discrimination and calibration performance of the XGBoost model. Plot (**A**) showed the ROC curves of the XGBoost model in the training set and validation set, respectively (AUC = 0.7854 versus 0.7941). Calibration curves of the XGBoost model in the training set (**B**) and validation set (**C**). AUC, area under the curve; ROC, receiver operating characteristic. XGBoost, extreme gradient boosting

The discrimination and calibration of the LASSO model and the XGBoost model in the training set and validation set were shown in Figs. 4A-C and 5A-C, respectively. In the training set, the AUC of LASSO model and XGBoost model were 0.7879 (0.7627–0.8132) and 0.7854 (0.7599–0.8109) respectively. In the validation set, the AUC of LASSO model and XGBoost model were 0.7994 (0.7618–0.8369) and 0.7941 (0.7560–0.8321), respectively. As shown in Figs. 4B, C, and 5B, C, in the calibration curves of the training set and the validation set of the same model, it can be seen that the prediction models had a strong concordance performance in both sets.

**Fig. 6:**
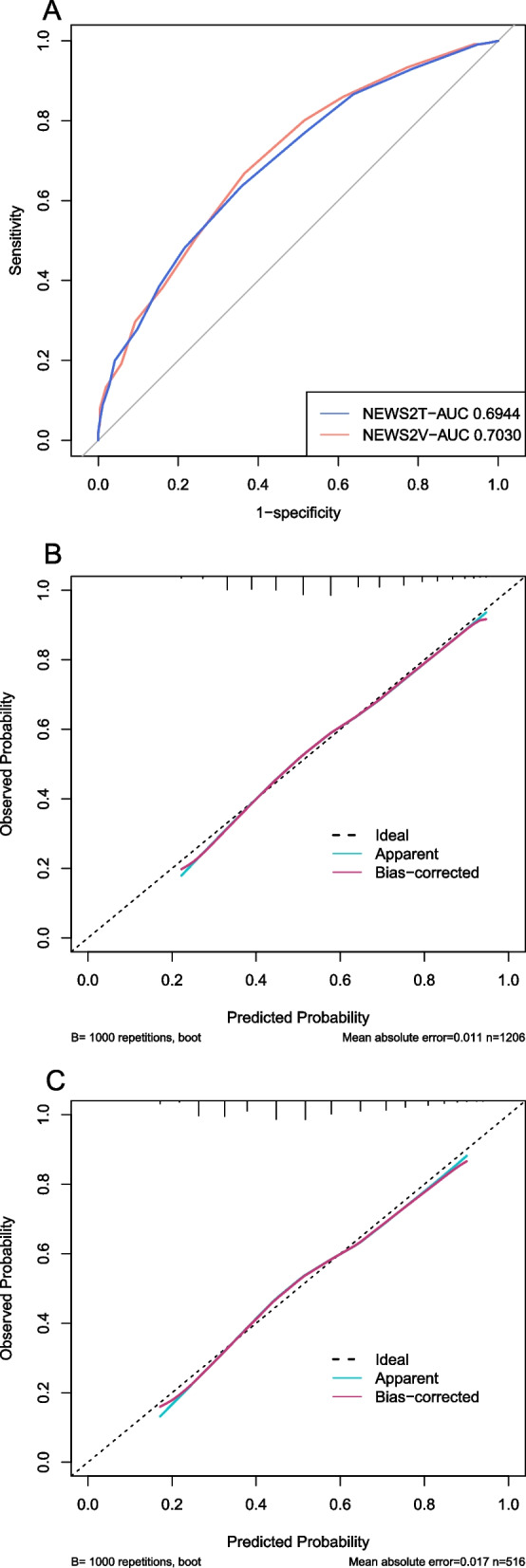
The discrimination and calibration performance of NEWS 2 model. Plot (**A**) showed the ROC curves of the NEWS 2 model in the training set and validation set, respectively (AUC = 0.6944 versus 0.7030). Calibration curves of the NEWS 2 model in the training set (**B**) and validation set (**C**). AUC, area under the curve; NEWS 2, the national early warning score 2; ROC, receiver operating characteristic

The NEWS 2 based on RR, SPO_2_, SBP, pulse rate, level of consciousness or new confusion, temperature to predict the risk of in-hospital mortality for patients in ICU with CA. We calculated the NEWS 2 for all study patients. The ROC curve and calibration curve of NEWS 2 in the training set and verification set were shown in Fig. 6A-C. The AUC of the training set and verification set were 0.6944 (0.6651–0.7237) and 0.7030 (0.6588–0.7472), respectively.

**Fig. 7:**
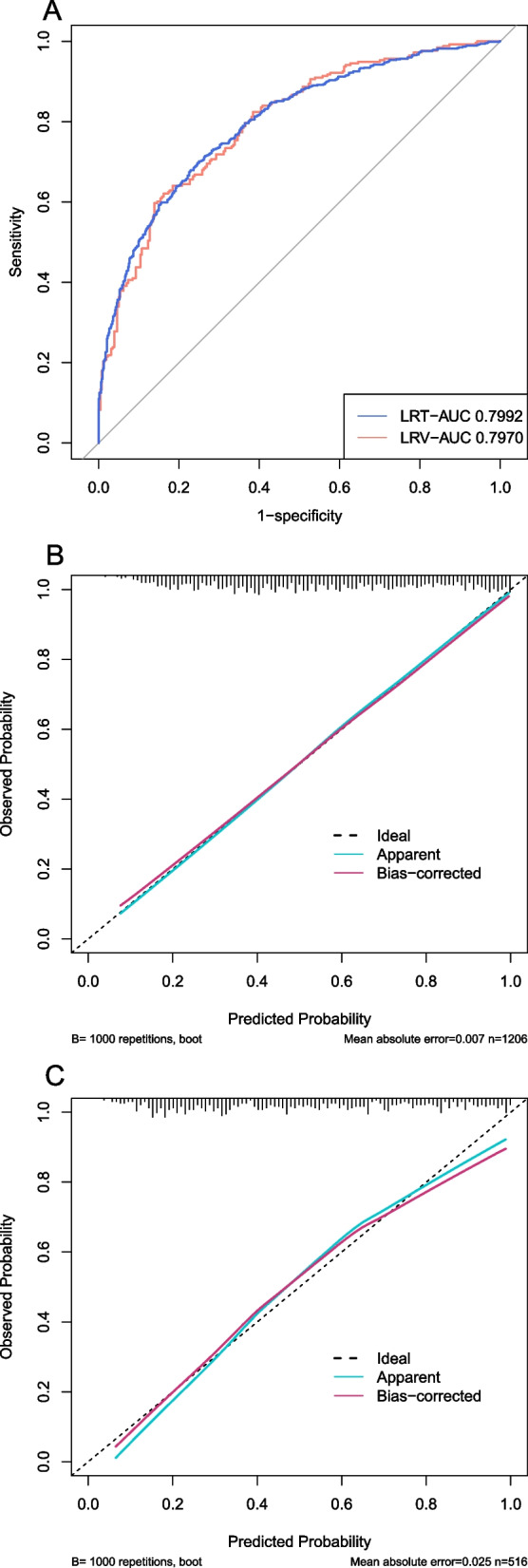
The discrimination and calibration performance of LR model. Plot (**A**) showed the ROC curves of the LR model in the training set and validation set, respectively (AUC = 0.7992 versus 0.7970). Calibration curves of the LR model in the training set (**B**) and validation set (**C**). AUC, area under the curve; LR, logistic regression; ROC, receiver operating characteristic

The ROC curve and calibration curve of LR model in the training set and verification set were shown in Fig. 7A-C. The AUC of the training set and verification set were 0.7992 (0.7746–0.8238) and 0.7970 (0.7592–0.8348), respectively.

**Fig. 8:**
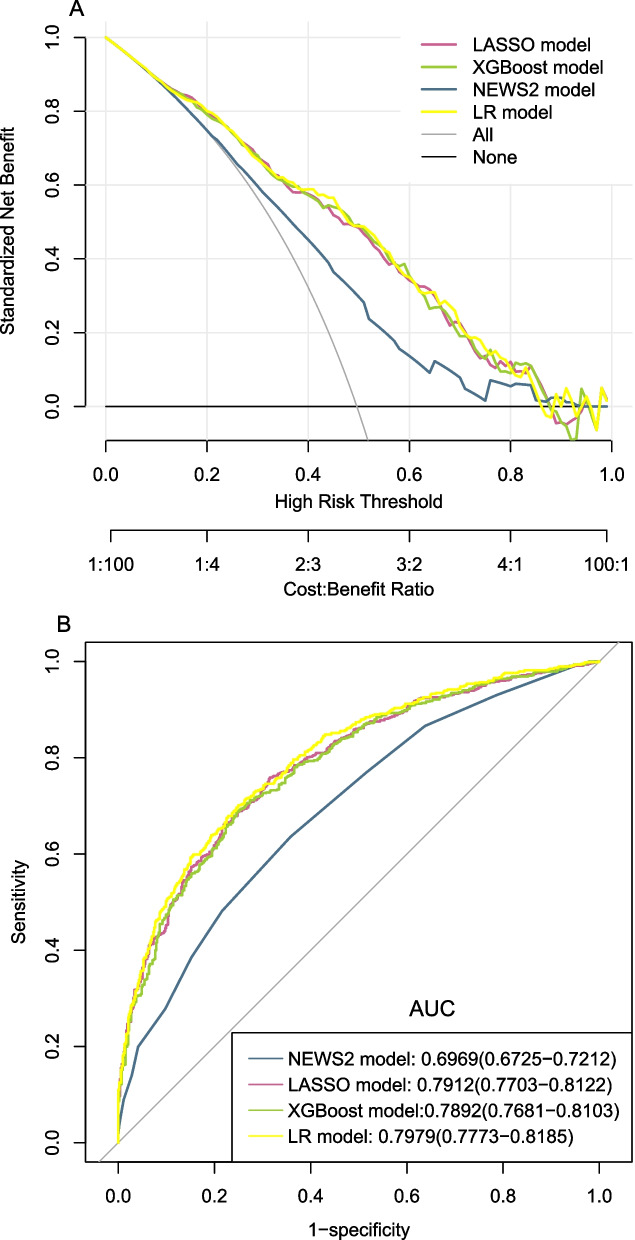
**A** Decision curve analysis for LASSO model, XGBoost model, LR model and NEWS 2 model. The y-axis measures the net benefit. The red line represents the LASSO model. The green line represents the XGBoost model. The yellow line represents the LR model. The blue line represents the NEWS 2 model. The grey line represents the assumption that all patients die in the hospital. The black line represents the assumption that no patients die in the hospital. **B** The comparison of ROC curves for LASSO model, XGBoost model, LR model and NEWS 2 model. LASSO, least absolute shrinkage and selection operator; LR, logistic regression; NEWS 2, the national early warning score 2; ROC, receiver operating characteristic; XGBoost, extreme gradient boosting

The DCA for the LASSO model, the XGBoost model, LR model and the NEWS 2 model were presented in Fig. 8A. It can be seen that when the threshold probability was 0.18 to 0.86 in the four models, the models added more net benefit than the ‘All’ or ‘None’ scheme.

### Model comparison

We compared the AUC of the LASSO, XGBoost, LRand NEWS 2 model in our total study population to assess the predictive effectiveness of the four models. Figure 8B showed that the AUC for the LASSO model, XGBoost model, LR model and NEWS 2 model were 0.7912(0.7703–0.8122), 0.7892(0.7681–0.8103), 0.7979(0.7773–0.8185) and 0.6969 (0.6725–0.7212), respectively, which were confirmed to be 0.7873, 0.7845, 0.7921 and 0.6969 via bootstrapping validation(repeat = 1000). By comparing the AUC values, the predictive effectiveness of the LASSO model,the XGBoost model and LR model were both significantly better than the NEWS 2 model (*p* < 0.001). And there was no statistical significance difference between the LASSO model, the XGBoost model and LR model (*p* > 0.05) (Table 4).

**Table 4:** Pairwise comparison of AUC for LASSO model, XGBoost model, LR model and NEWS 2 model

Comparison between models	Z statistic	95% Confidence interval	Significance level (*p* value)
LASSO model vs XGBoost model	0.8	-0.003 to 0.007	0.40
NEWS 2 model vs LASSO model	-10	-0.114 to -0.075	< 0.001
NEWS 2 model vs XGBoost model	-9	-0.112 to -0.073	< 0.001
LASSO model vs LR model	-1	-0.016 to 0.003	0.20
XGBoost model vs LR model	-2	-0.019 to 0.002	0.10
NEWS 2 model vs LR model	-10	-0.121 to -0.081	< 0.001

*AUC* Area under curve, *LASSO* Least absolute shrinkage and selection operator, *LR* Logistic regression, *NEWS 2* the national early warning score 2, *XGBoost* extreme gradient boosting. *P* < 0.05 was statistically significant

**Fig. 9:**
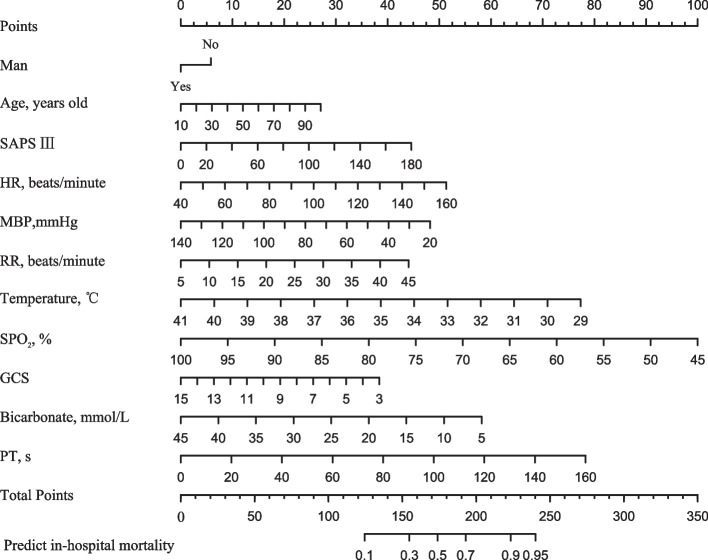
Predict in-hospital mortality nomogram. GCS, Glasgow coma scale; HR, heart rate; MBP, mean blood pressure; PT, prothrombin time; RR, respiratory rate; SAPS III, simplified acute physiology score; SPO2, saturation pulse oxygen

In the LASSO model, 11 variables were included, and there were 9 variables in the XGBoost model, while there were 15 variables in the LR model. LR model had more variables and a smaller threshold range compared to XGboost model and LASSO model. Although the XGboost model was more concise, the net benefit of the LASSO model was higher than the XGBoost model within the threshold range of 0.6–1.0. We believed that higher net benefit was more beneficial for patients with CA. Therefore, we chose the LASSO model as the final model, and represented by the nomograph in Fig. 9. The nomograph used some parallel lines with scales to estimate the probability of occurrence of each risk factor. The score of each risk factor can be calculated, and then the probability of occurrence to the total score of all risk factors can be calculated, which is the probability of occurrence of this event.

## Discussion

CA is a major public health event with a high mortality rate. Early and accurate prediction of in-hospital mortality of patients with CA can give clinicians more time to provide individualized treatment strategies, and play an important role in rational planning of medical resources and personnel scheduling. Many studies had analyzed the short-term and long-term survival rates of OHCA and IHCA respectively. Recently, more and more studies established prediction models for in-hospital patients with CA [31–33].

With the advancement of electronic medical records and artificial intelligence, ML algorithms have become more widely utilized in individualized medicine to assist clinical decision-making. Therefore, we used the data from MIMIC-IV database to screen the independent risk factors of in-hospital mortality with CA patients by ML algorithm, and then obtained a predictive nomograph by logistic regression analysis.

In our study, the in-hospital mortality is 52.4%, which is significantly lower than other study population [2, 4, 34]. The lower mortality rate may be related to the small sample size and the location of CA. The age, HR, RR, bicarbonate, SPO_2_, temperature, SAPS III score and GCS score were included in the XGBoost and LASSO multiple regression equations. The HB were included in the XGBoost multiple regression equation; Meanwhile, the man, PT and MBP were included in the LASSO multiple regression equation. Consistent with previous studies, our study also took age, man, HR, SPO_2_, and lower MBP values as independent predictors [32, 35, 36].

After CA, the occurrence of tissue ischemia and hypoxia will lead to the decrease of pH value and increase of lactate value. In OHCA patients, the pH value was related to the neurological state of the patient at discharge, which may help predict the adverse neurological state of the patient at discharge [37, 38]. However, some studies had shown that dynamic monitoring of pH may be more meaningful for the prognosis of the nervous system [39]. Moreover, the prognosis of the nervous system was not consistent with the in-hospital survival rate. Lactate was also an indicator that can affect the prognosis of CA patients [40]. However, some studies had pointed out that in addition to the absolute value of lactate, the change rate of early lactate value also had important prognostic significance [41, 42]. In our study, pH and lactate were not included in the final model due to the inconsistent sample collection time of all patients and the possible bias caused by the longtime span of the database.

Several prognostic scoring systems had been developed to predict the in-hospital mortality of ICU patients [43]. SAPS III was a more modern mortality prediction model that used a larger cohort that included the first from outside North America and Europe and utilized new computer-intensive analysis methods [44]. One study showed that the SAPS III did not predict mortality in patients admitted to ICU after CA [45]. SAPS III was also considered as a reliable, simple and easy to use prognostic model in clinical practice [46]. In our study, SAPS III was included in the final model as an independent predictor.

Targeted temperature management is the sole intervention for improving neurological outcomes in the post return of spontaneous circulation phase of care, which has been considered a standard of care treatment for over almost two decades and has been included in the International Post-Cardiac Arrest Guidelines [47, 48]. However, in our study, the effect of temperature was opposite to targeted temperature management, which may be because low body temperature was due to poor peripheral circulation, rather than hypothermia treatment.

During CA, metabolic acidosis is caused by hypoxia-induced anaerobic metabolism and decreased metabolic acid excretion due to renal insufficiency [49]. The lower the bicarbonate, the more serious the metabolic acidosis. In our study, bicarbonate was included in our model as an independent predictor.

PT shows the status of exogenous coagulation system. PT prolongation was prevalent in critically ill patients and was independently associated with higher ICU mortality [50]. In other studies, prolonged PT was also associated with increased mortality [51, 52]. In our study, PT was an independent predictor of in-hospital mortality in patients with CA.

NEWS 2 has clinically significant in predicting the incidence of CA within 24 h, ICU occupancy and mortality [53, 54]. Compared with the NEWS 2 model, XGBoost model,LASSO model and LR model showed the advantages of prediction effect in our study. The three models showed good discrimination and calibration abilities in the training set and validation set. To obtain a wider threshold range and a greater net benefit, we selected the LASSO model to develop our predict nomogram.

A lot of variables were reported to correlate with mortality in CA patients, such as hypertension, DM, the use of vasopressor [13, 40, 55, 56]. Nevertheless, our study showed that hypertension, DM and the use of epinephrine and dopamine were not predictors of in-hospital mortality of ICU-admitted CA patients, which may be attributed to our relatively small sample size, and the difference in amount and duration of use vasopressors.

Our study had several limitations. Firstly, as this was a retrospective study, we were unable to avoid selection bias. Secondly, as a single center study with the earliest cases from almost 20 years ago, the treatment and care of CA had been inconsistent with current standards, which requires a multicenter registration and prospective study to verify. Thirdly, our cases were all adult CA patients in ICU. Whether the results of this study can be applied to other populations needs further research on more patients in different clinical environments to confirm our results. Fourthly, limited by the contents of the MIMIC-IV database, incomplete recorded data was not included in the analysis, moreover, we cannot distinguish the source of patients in this study was IHCA or OHCA. Fifth, due to the small number of patients with CA, in order to ensure the accuracy of the model, we used survival rate as an outcome indicator rather than neurological prognosis. In the future, it may be more meaningful to study neurological prognosis as an outcome when the sample size is sufficient.

## Conclusion

We developed a predictive nomogram for in-hospital mortality of CA patients in ICU, which included variables that can be routinely collected during hospitalization in ICU. With a high AUC of 0.7912 (95% CI 0.7703–0.8122), a wide net benefit threshold range (0.2–1.0) and high net benefit, this nomogram may be widely used in clinical decision-making.

## Supplementary Information

**Additional file 1.**

## Data Availability

The datasets used and analyzed during the current study are available from the corresponding author on reasonable request.

## Abbreviations

AUC: Area under curves
BUN: Blood urea nitrogen
CA: Cardiac arrest
COPD: Chronic obstructive pulmonary disease
CHF: Congestive heart failure
DCA: Decision curve analysis
DM: Diabetes mellitus
DBP: Diastolic blood pressure
XGBoost: Extreme gradient boosting
GCS: Glasgow Coma Scale
HR: Heart rate
HCT: Hematocrit
HB: Hemoglobin
pH: Hydrogen ion concentration
IHCA: In-hospital cardiac arrest
ICU: Intensive care unit
IQR: Inter quartile ranges
INR: International normalized ratio
LASSO: Least absolute shrinkage and selection operator
LR: Logistic regression
ML: Machine learning
MBP: Mean blood pressure
MIMIC-IV: Medical Information Mart for Intensive Care IV
NEWS 2: National Early Warning Score 2
OHCA: Out-of-hospital cardiac arrest
PT: Prothrombin time
RR: Respiratory rate
ROC: Receiver operating characteristic
SPO_2_: Saturation pulse oxygen
SOFA: Sequential Organ Failure Assessment
SAPS III: Simplified Acute Physiology Score III
SBP: Systolic blood pressure
WBC: White blood cells
