# Supplementary material for: Prediction model of in-hospital mortality in intensive care unit patients with cardiac arrest: a retrospective analysis of MIMIC -IV database based on machine learning
Source: BMC Anesthesiol. 2023 May 25;23:178. doi: 10.1186/s12871-023-02138-5 (PMC10210383; doi:10.1186/s12871-023-02138-5)
Supplement: Supplementary file 1 — Additional file 1. [file 12871_2023_2138_MOESM1_ESM.docx]

| Supplementary Table 1: Missing value details | | | | | |
| --- | --- | --- | --- | --- | --- |
| Variates | Number of missing values | Percentage of missing values | Variates | Number of missing values | Percentage of missing values |
| Heart rate | 4 | 0.23% | Sodium | 24 | 1.39% |
| Respiratory rate | 5 | 0.29% | Chloride | 25 | 1.45% |
| MBP | 6 | 0.39% | Potassium | 25 | 1.45% |
| Hematocrit | 8 | 0.46% | Bicarbonate | 26 | 1.51% |
| Platelets | 8 | 0.46% | Anion gap | 27 | 1.57% |
| BUN | 8 | 0.46% | Glucose | 29 | 1.68% |
| Hemoglobin | 9 | 0.52% | PT | 48 | 2.79% |
| Creatinine | 9 | 0.52% | INR | 50 | 2.90% |
| WBC | 10 | 0.58% | Calcium | 74 | 4.30% |
| SBP | 12 | 0.70% | Temperature | 251 | 14.58% |
| DBP | 12 | 0.70% | pH | 297 | 17.28% |
| SPO_2_ | 16 | 0.93% | Lactate | 420 | 24.39% |

BUN, blood urea nitrogen; DBP, diastolic blood pressure; INR, international normalized ratio; MBP, mean blood pressure; pH, hydrogen ion concentration; PT, prothrombin time; SBP, systolic blood pressure; SPO_2_, saturation pulse oxygen; WBC, white blood cells;
